# V(D)J Rearrangement Is Dispensable for Producing CDR-H3 Sequence Diversity in a Gene Converting Species

**DOI:** 10.3389/fimmu.2018.01317

**Published:** 2018-06-11

**Authors:** Philip A. Leighton, Jacqueline Morales, William D. Harriman, Kathryn H. Ching

**Affiliations:** Ligand Pharmaceuticals, Emeryville, CA, United States

**Keywords:** CDR-H3 repertoire, somatic hypermutation, transgenic chickens, gene conversion, V(D)J rearrangement, human monoclonal antibodies

## Abstract

An important characteristic of chickens is that the antibody repertoire is based on a single framework, with diversity found mainly in the CDRs of the light and heavy chain variable regions. Despite this apparent limitation in the antibody repertoire, high-affinity antibodies can be raised to a wide variety of targets, including those that are highly conserved. Transgenic chickens have previously been generated that express a humanized antibody repertoire, with a single framework that incorporates diversity by the process of gene conversion, as in wild-type chickens. Here, we compare the sequences and antibodies that are generated purely by gene conversion/somatic hypermutation of a pre-rearranged heavy chain, with the diversity obtained by V(D)J rearrangement followed by gene conversion and somatic hypermutation. In a gene converting species, CDR-H3 lengths are more variable with V(D)J rearrangement, but similar levels of amino acid diversity are obtainable with gene conversion/somatic hypermutation alone.

## Introduction

As in all higher vertebrates, a critical checkpoint in B cell development in chickens is in-frame V(D)J rearrangement, leading to expression of a functional B cell receptor complex at the cell surface (1–3). The process of rearrangement in chickens utilizes the same recombination signal sequences and enzymes as in mammals, recombining V, D, and J genes into functional V regions (4–8). The main difference in chickens is that in both the light and heavy chain loci, there is only a single germline V gene and a single germline J gene, and in the heavy chain, a cluster of highly similar D segments (5, 8), rather than the large number of diverse V, D, and J genes in humans. The rearrangement process thus produces very little sequence diversity in the initial B cell repertoire. Imperfect joins and exonucleolytic chewing back in the V-J and V-D-J junctions can generate some diversity, but chicken B cells do not express TdT (9), so there are no N-additions in CDR-H3, and in general, the immunoglobulin diversity produced by the gene rearrangement process is minimal (5, 10, 11). To produce a diverse repertoire, chickens employ a process of gene conversion in which upstream pseudogenes in the light and heavy chain loci serve as sequence donors to mutate the expressed, functional V (8, 11–13). These pseudogenes do not contain promoters or recombination signal sequences, so they cannot be expressed themselves, but their sequences are incorporated in segments of varying length into the single functional V. Multiple rounds of overlapping gene conversion from different pseudogenes in the pool lead to a highly diverse naïve repertoire. In addition to gene conversion, non-templated somatic hypermutation also contributes to repertoire diversity (14–16). Despite the limitations of V(D)J rearrangement in chickens, CDR-H3s exhibit length and sequence diversity comparable to that in mammals (1, 17). The function of the chicken Ds in V(D)J rearrangement may be more related to providing intra-CDR-H3 disulfide bridges for stabilization of antigen-binding loops (17), since most Ds encode a single cysteine residue and D-D joins would thus encode paired cysteines. Diversity in chicken CDR-H3s comes from gene conversion/somatic hypermutation, rather than the rearrangement process itself.

We have engineered transgenic chickens to produce a human variable region antibody repertoire (OmniChickens™) (18, 19). From a pharmaceutical standpoint, the use of a single V framework in chickens could be an advantage since we may select a preferred FR with optimal manufacturing and developability characteristics (20). Transgenes can be designed such that diversity is focused in the CDRs, while maintaining germline or near-germline FR sequences. Our strategy is to retain the advantage of the chicken B cell system for incorporating diversity, using a single human framework with upstream human-based pseudogenes, instead of inserting a large human genomic fragment (carrying multiple human V, D, and J genes) which may not be regulated properly in the chicken. For the single human framework to provide therapeutic candidates to any potential target, the level of diversity produced by the transgene must be sufficient. Our hypothesis was that gene conversion alone could generate sufficient diversity in human CDR-H3, since that is essentially the normal process in chickens. To test this idea, we produced two lines of transgenic chickens: one carrying a pre-rearranged, functional VH region that relies entirely on gene conversion/somatic hypermutation to produce diversity, and one which undergoes V(D)J rearrangement first to produce a functional VH region, thereby potentially increasing the level of CDR-H3 diversity, followed by gene conversion/somatic hypermutation. In a gene converting animal, is there any advantage to providing D gene diversity during the recombination process, or can the CDR-H3 repertoire be completely produced by gene conversion?

## Materials and Methods

**Figure 1:**
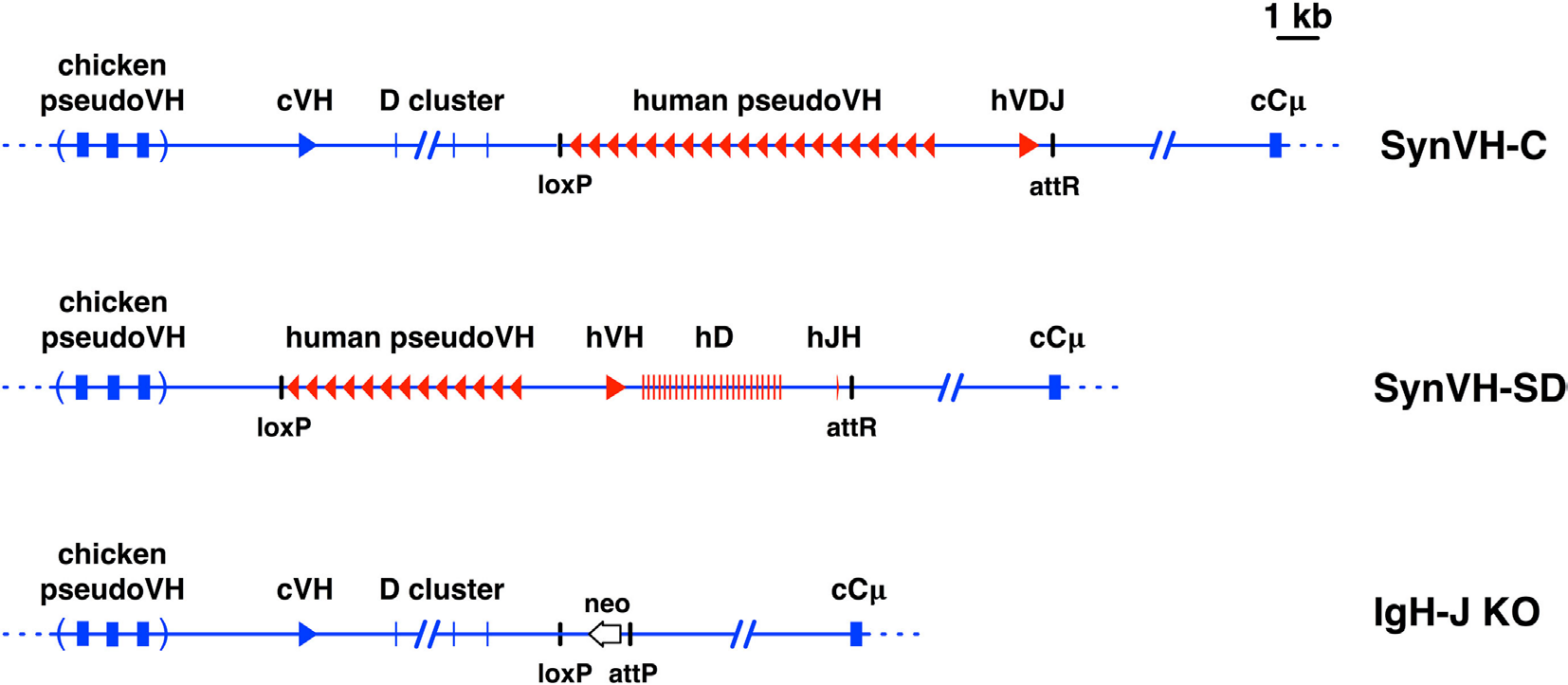
Diagrams of pre-rearranged and rearranging human VH genes in the chicken. Scale diagrams of SynVH-C (pre-rearranged) and SynVH-SD (rearranging) transgenes and the heavy chain knockout (IgH-J KO). Human sequences are shown in red and chicken in blue. Top line, the SynVH-C transgene consists of a pre-rearranged human V region (hVDJ) with an upstream array of human pseudogenes. This diagram was previously published (18). The human V region splices to the downstream chicken constant regions (only Cμ is shown). The chicken germline V and D genes, and pseudogene array, are upstream of the human V genes. Precise mapping of chicken pseudogenes is not shown (as indicated by parentheses), but the distance to the functional chicken V is accurate. Remnant loxP and attR sites from the insertion event are shown. Middle line, the SynVH-SD transgene contains a single human germline VH3-23 gene, single JH6 gene, and 24 unique human Ds. All intervening sequences and recombination sites are from the chicken heavy chain locus (shown in blue). Upstream of the human germline V gene is an array of human-based pseudogenes. The upstream chicken germline V and D genes are deleted, but the chicken pseudogenes are still present. Bottom line, structure of the chicken heavy chain knockout (21). The genotype of the OmniChickens in this study was SynVH-C or SD “knock-in”/IgH knockout. The single chicken JH gene was replaced with a promoterless neo gene. The attP site adjacent to the neo gene is where the SynVH-C and SynVH-SD constructs were inserted, followed by Cre-lox recombination to remove selectable markers and plasmid backbone sequences.

The pre-rearranged VH region construct, SynVH-C, was previously described (18). The V(D)J rearranging construct, SynVH-SD, was made by gene synthesis of several parts followed by ligation. The V, D, and J regions were assembled as follows. The human germline VH3-23*01 gene was the single V gene used, and the JH6 gene was the single J gene. 24 non-redundant human Ds were flanked by recombination signal sequences and intervening regions from the chicken D locus (these spacers were about 100–200 bp each). Cysteine codons in the human D2 family were mutated to encode tyrosine (seven instances) or tryptophan (two instances). The rearranging elements were cloned with the chicken VH promoter to drive expression of the heavy chain, and a short section of the chicken J-C intron for splicing to the endogenous constant regions was included. The human pseudogenes contained FRs from the human germline VH3-23 gene and CDRs 1 and 2 from the VH3 germline gene family. 13 pseudogenes were designed. Spacer sequences between each pseudogene were from the chicken pseudogene region, but did not include chicken V sequences themselves. An attB site for insertion into the attP site targeted to the chicken heavy chain locus was included (21, 22), and a loxP site for later recombination with loxP sites in the target genome. The SynVH-SD construct was transfected into the heavy chain attP-containing cells. In these cells, a loxP site had previously been inserted by CRISPR-mediated targeting upstream of the chicken VH gene (22). After insertion of the SynVH-SD transgene, a second loxP site was brought in by the SynVH-SD transgene, in the same orientation as the first loxP site, directly upstream of the human pseudogene array. Breeding to Cre hens removed all of the DNA between the loxP sites, which included the chicken VH and D genes and the selectable markers used during the transfections, leading to the structure shown in Figure 1. For the light chain, all of the OmniChickens used in this study expressed a human V-kappa light chain, from construct SynVK-CK (18). The light and heavy chains in these birds consisted of human variable regions and chicken constant regions. At the heavy chain locus, the transgenes were heterozygous in all cases, with the knockout on the other allele, giving the genotype *IgH^SynVH^*/*IgH^JH-KO^*. At the light chain locus, the genotype was always *IgL^SynVK-CK^*/*IgL^VJC-KO^*.

OmniChickens containing SynVH-C were immunized as described (18). SynVH-SD birds were immunized either with progranulin (PGRN) protein or with a combination of DNA and PGRN protein, on a schedule similar to the SynVH-C birds. Monoclonal antibodies were identified by gel-encapsulated microenvironment (GEM) screening of single B cells from spleens of immunized birds (18, 23).

Spleen lymphocytes were prepared by Ficoll density centrifugation. Total RNA from spleen lymphocytes (approximately 10^7^) was extracted using RNeasy (Qiagen). 10–15 ng total RNA was used in a OneStep Ahead (Qiagen) proofreading polymerase reaction using primers chVH-F9 (5′-CACCAGTCGGCTCCGCAACCATG-3′) and cIgY-NGS-R (5′-gggcgatgtggggctcgc-3′). Amplicons of approximately 450 bp were obtained and sequenced at ABM (Richmond, BC, Canada). ABM performed paired end merging, cluster analysis, matching of sequences to the previously identified mAb sequences, and CDR-H3 length determination.

Sequences were aligned and analyzed using DNAstar software. Excel macros were downloaded from the web site of Annemarie Honegger (University of Zurich) and used for calculating amino acid frequencies.

Animal experiments were done in accordance to Ligand Pharmaceuticals IACUC approved protocols and under supervision of the IACUC committee.

## Results

We analyzed human VH sequences from chickens with two different human heavy chain transgenes. One of these transgenes, SynVH-C, contained a pre-rearranged functional V region (18), and the other, SynVH-SD, contained germline V, D, and J elements that undergo rearrangement in B cells (Figure 1). The V region in SynVH-C was obtained from screening a human library and it contained a rearranged VH3-23/D1/JH4 region, with nine framework (FR) changes relative to the germline VH3-23 gene. The SynVH-SD construct contained a single germline V gene, VH3-23, all of the human D elements, and a single JH6 gene. The D elements were separated by intervening sequences from the chicken D locus, including the highly conserved recombination signal sequences. All of the other non-coding sequence (promoter and introns) in both constructs was derived from the chicken heavy chain locus for optimal transcriptional and post-transcriptional regulation. The transgene constructs were inserted into the endogenous heavy chain locus *via* a combination of gene targeting and integrase-mediated insertion (18, 22). The human V regions in both transgenes splice to the endogenous downstream chicken constant regions.

Both of the transgenes contained upstream human-based pseudogenes which can participate in gene conversion of the functional human V (8, 11). The pseudogenes were designed with diversity mainly in the CDRs (Figure S1 in Supplementary Material), although some of the SynVH-C pseudogenes also contain changes in the frameworks. These pseudogenes were designed *de novo* and bear no relation to the V gene pseudogenes resident in the human genome. Two different approaches to pseudogene CDR design were taken. In SynVH-C, CDRs were derived from naturally occurring CDR sequences found in human sequence databases of expressed sequences (ESTs). All three CDRs were included in the pseudogenes, and the 3′ end of each pseudogene includes CDR3 but does not extend into FR4 or include the invariant Trp-118 residue marking the border of CDR3. Between CDR3 of one pseudogene and the beginning (FR1) of the next pseudogene, spacer sequences of 100 bp were placed. In SynVH-SD, the CDRs were from the germline human VH3 family members, CDR1 and 2, with no specific CDR3 sequences in the pseudogenes, since germline V genes do not contain CDR3. Downstream of FR3 were placed the spacer sequences, which are diverse sequences that could potentially be used in gene conversion even though they are not derived from human CDR3s (Figure S1 in Supplementary Material). After insertion, the chicken pseudogene array was still present upstream in both cases. In the case of SynVH-C, the single chicken germline VH and D cluster that normally undergo VDJ rearrangement were still present, but they should not be able to recombine into a functional V region because there is no germline JH region, and the human V region downstream is fully rearranged. In the SynVH-SD transgene, the chicken V and D genes were deleted (22) to eliminate the possibility that the chicken genes would directly recombine with the human JH gene and displace the human V and D genes. The only JH region present in the genome for both lines of birds was the human JH in the SynVH transgene (Figure 1).

Heavy chain V regions were sequenced in bulk by NGS amplicon sequencing from spleen lymphocyte populations of nine immunized birds (see below for details of immunization). The VH regions were amplified from lymphocyte RNA following reverse transcription, using a forward primer in the 5′ UTR and a reverse primer in the IgY constant region CH1 domain, and sequenced by MiSeq (Applied Biological Materials, Canada). The primers used to amplify VH regions were chosen so that they would amplify the human V region or the chicken V region, should it be expressed. In the human transgenes, the only sequence that is human is the V region coding sequence. All of the non-coding sequences (the 5′ UTR, introns, etc.) and the constant region coding sequences were chicken sequence in both SynVH constructs. Paired-end reads were assembled and translated to provide theoretical protein sequence. The number of reads, unique nucleotide, and protein sequences from each bird are given in Table 1. The most common sequence in each bird ranged in the number of times it was found from about 6,700 to 89,000. For some of the analysis, the top 1,000 most common unique sequences from each sample were used, which represented 34–62% of the total sequence data from each sample.

**Table 1:** Summary of sequencing data.

Sample	% merged by FLASH	# unique nucleotide sequences	# unique peptide sequences	# unique pep at 2× depth	# hits of top sequence	Top 1K % of total sequence
**SynVH-C**						
23806	90.56	676,328	434,570	41,751	41,745	41.27
23824	90.35	940,993	632,148	81,377	28,173	40.06
24317	92.17	930,254	637,959	60,330	17,049	53.55
26934	91.00	791,431	540,981	70,701	20,487	40.62
27022	88.80	925,088	582,374	92,873	20,228	41.81
27023	90.66	1,006,989	636,318	68,348	26,221	62.30
**SynVH-SD**						
29406	90.27	387,390	254,102	34,733	6,706	36.00
29407	90.18	696,921	409,293	56,921	89,009	46.85
29409	89.43	939,220	571,584	76,879	17,450	34.01

*Samples from six SynVH-C and three SynVH-SD birds are listed by their bird number. The numbers of unique nucleotide and protein sequences are given, and the number of unique peptide sequences that were sequenced two or more times (2× depth). The proportion of the total sequence reads represented by the top 1,000 sequences is given in the last column*.

### V Region and Signal Peptide Usage

We started analyzing the VH regions from the nine birds by determining whether the expressed sequences were fully human, as expected, or contained any chicken sequence (Table 2). All of the V regions from SynVH-SD were human sequence, which was expected since the endogenous chicken V region was deleted upstream of the SynVH-SD insertion. Unexpectedly, about 5% of the sequences from the SynVH-C transgene were chicken VH regions. These sequences were comprised of chicken CDRs 1–2 and FRs 1–3, fused to CDR3 and the human J sequence, which is the only possibility since the only JH sequence present in the genome of the OmniChickens is the human J on the SynVH transgene. CDR-H3 appeared to be human, since there are no non-canonical cysteines, which would be expected in chicken CDR-H3s (17). There were two potential sources of chicken VH sequences in the expressed antibody repertoire in SynVH: gene conversion from the chicken pseudogenes, which are still present upstream of the human pseudogenes, or gene replacement of the human functional V with the chicken functional V by a secondary rearrangement type mechanism in which the chicken V gene rearranged with the human V gene *via* a cryptic recombination signal sequence at the 3′ end of the V gene (24–27). It seems most likely that these chicken V regions came from secondary rearrangement, fusing in frame at the human FR3-CDR3 junction in the transgene, thereby deleting the human V gene. The main evidence to favor gene replacement over gene conversion is that the signal peptide sequence was also chicken, whereas the pseudogenes do not contain signal peptide sequences and thus could not mutate the human signal peptide to a chicken signal peptide sequence. In addition, if gene conversion could replace the expressed V region with the chicken V region, then one might expect the same occurrence in the SynVH-SD sequences, which also contains the upstream pseudogenes, but no V replacement was observed in SynVH-SD. The functional chicken V and D cluster have been deleted upstream of the SynVH-SD transgene which precludes the possibility of gene replacement.

**Table 2:** SynVH-C but not SynVH-SD showed gene replacement by chicken VH, whereas both had changes in the signal peptide.

	Chicken VH		Human VH		huJH
			
	Ch sig pep	Hu sig pep	Ch sig pep	Hu sig pep	
SynVH-C	333 (5.6%)	5 (0.08%)	318 (5.3%)	5,331 (88.9%)	6,000 (100%)
SynVH-SD	0	0	613 (20.5%)	2,384 (79.5%)	3,000 (100%)

*The number (and percentage of total sequences from each transgene) of chicken and human VH regions are shown. Data from the six SynVH-C samples and three SynVH-SD samples were combined (total, 6,000 sequences from SynVH-C birds and 3,000 sequences from SynVH-SD birds). In the first set of columns are shown the sequences containing the chicken VH, split into those with a chicken signal peptide (Ch sig pep) or human signal peptide (Hu sig pep). In the second set of columns are the sequences with the human VH, similarly split for sequence of signal peptide. All of the sequences had the human JH (right column). The total number of VH regions is slightly less than the total number of sequences because a few sequences had VH deletions*.

**Figure 2:**
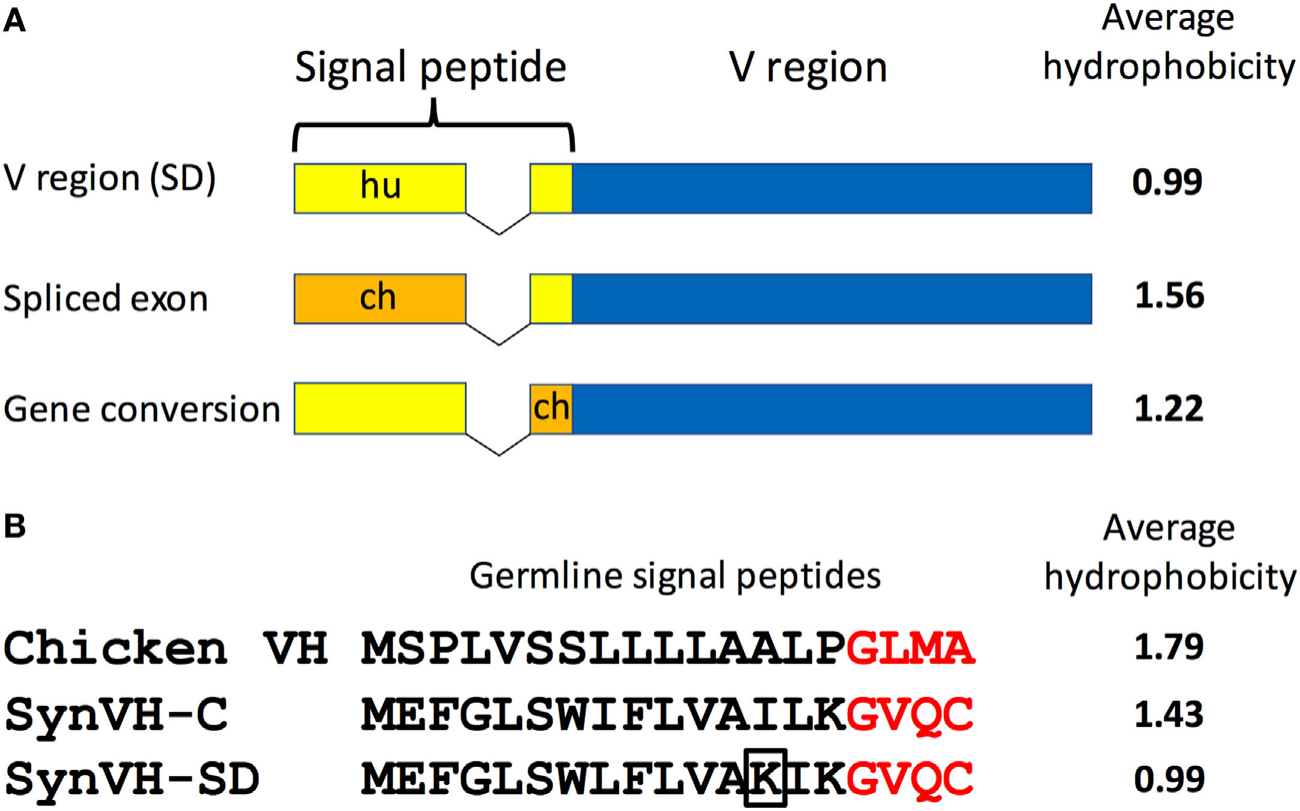
The SynVH-SD signal peptide is less hydrophobic than that of SynVH-C and undergoes changes to become more hydrophobic. **(A)** Diagram of the signal peptide changes observed in OmniChickens. The signal peptide can be changed to chicken sequences either by splicing of the chicken signal peptide exon to the human V region exon or from gene conversion of the portion in the V region exon. Human signal peptide is shown in yellow, chicken in orange. The average hydrophobicity (Kyte–Doolittle, without normalization) is shown for each type of signal peptide. **(B)** Alignment of the germline signal peptide sequences from the WT chicken VH gene, SynVH-C, and SynVH-SD. The four amino acids encoded in the V region exon are in red. The lysine residue that is often mutated to a hydrophobic residue in SynVH-SD is boxed. The average hydrophobicity is indicated at right.

We also noticed that the signal peptide did not always match the sequence that was introduced in the transgene construct. Although 80–90% of the sequences in SynVH-C and SynVH-SD transgenes contained the human signal peptide fused to human V sequences, sequence variation in the signal peptide was found. We observed three patterns of changes in the signal peptide. In the first pattern, the intact chicken signal peptide exon was spliced in-frame directly to the human V region exon, which was found in approximately 20% of the sequences in SynVH-SD and 5% in SynVH-C (Figure 2; Table 2). The higher frequency of signal peptide changes in SynVH-SD suggests that there is a selection for such changes. Comparison of the signal peptide sequences in SynVH-C, SynVH-SD, and chicken VH showed that SynVH-SD contains a Lys residue in the central hydrophobic domain, where the SynVH-C contains Ile, which makes it less hydrophobic than either the SynVH-C signal peptide or the chicken VH signal peptide and potentially a less efficient signal peptide (28) (Figure 2). The SynVH-SD signal peptide is identical to the human germline VH3-23 gene, whereas the SynVH-C signal peptide contains the Ile mutation relative to the germline. In the second pattern of signal peptide changes, the portion of the signal peptide encoded in the first exon was human but the four amino acids of the signal peptide that are encoded in the V exon were chicken (Figure 2). This pattern was observed in about 15% of the SynVH-SD sequences, but never in SynVH-C. This region is present in some of the chicken pseudogenes (8), so it is likely that gene conversion was responsible for mutating this region of the signal peptide to chicken. The chicken-derived sequence in this region extended slightly farther in these instances, to the third amino acid of the mature VH region (see below). In the third pattern of signal peptide changes, the SynVH-SD sequences retained the full human signal peptide, but carried a point mutation in the Lys residue (Figure 2), changing it to a hydrophobic residue (Val, Ile, or Met). This pattern occurred in about 40% of the SynVH-SD sequences. All three of these types of changes increased the hydrophobicity of the signal peptide. The high prevalence of these different types of changes to the SynVH-SD signal peptide strongly indicates positive selection for these changes.

### Gene Conversion in SynVH-C and SynVH-SD by Chicken Pseudogenes Is Rare

The chicken pseudogene array is still present upstream in both SynVH-C and SynVH-SD transgenes (Figure 1). In SynVH-SD, the chicken pseudogenes are in closer proximity to the human functional V, immediately upstream of the human pseudogenes, whereas in SynVH-C, the chicken V and D genes are between the two pseudogene arrays. As discussed above, the signal peptide sequence adjacent to FR1 underwent gene conversion in SynVH-SD, so we were interested to know if further gene conversion of the human frameworks by chicken pseudogenes occurred. At the DNA level, the chicken germline VH gene is about 65% identical overall to the V genes in our transgenes. The longest stretches of homology are 11 bp, and mismatches of 1–6 bp are spread throughout the V regions. It is unclear whether this level and pattern of homology would be sufficient to enable gene conversion. To determine the functionally relevant levels of gene conversion that lead to changes in the protein sequence, FR1 and 3 were analyzed at the protein level for evidence of long stretches of chicken residue replacement (FR2 is too conserved between chicken and human to be able to unequivocally detect gene conversion events). Very low levels of gene conversion of the human FR1 and 3 in SynVH-C (0.07% in FR1 and 0.3% in FR3, in 5,652 sequences) and SynVH-SD (2% in FR1 but none in FR3) were observed (Table 3). The most common example of gene conversion was the signal peptide change in SynVH-SD, which occurred in about 15% of the sequences, suggesting that if the sequence changes were under selection, then gene conversion could be observed at a higher frequency. The low frequencies of gene conversion events by chicken pseudogenes in the human FRs suggest that they are rare, non-selected events. The slightly higher frequency (2%) in SynVH-SD FR1 could be a result of a continuation of gene conversion events that began in the signal peptide region at the 5′ end of the gene and continued into FR1. Another potential contributing factor could be the closer physical proximity on the chromosome of the human functional V to the chicken pseudogenes in SynVH-SD since the chicken V and D cluster were deleted in that transgene (Figure 1). In the antigen-specific mAb sequences derived from these birds (see below), no chicken-derived FR residues were seen in any sequence, which bolsters the idea that these rare gene conversion-derived sequences were not under selection nor were they necessary to produce antigen binders.

**Table 3:** Gene conversion of human FRs by chicken pseudogenes is rare.

Sample	Signal peptide/FR1^a^	FR1^b^	FR2	FR3	FR4	# human sequence
**SynVH-C**						
23806	0	2	ND	0	0	985
23824	0	1	ND	1	0	946
24317	0	1	ND	0	0	976
26934	0	0	ND	1	0	946
27022	0	0	ND	10	0	946
27023	0	0	ND	4	0	853
**SynVH-SD**						
29406	172	8	ND	0	0	1,000
29407	113	0	ND	0	0	1,000
29409	174	60	ND	0	0	1,000

*The number of gene conversion events observed in the human sequences from each bird is given. Gene conversion is defined as a long tract of sequence (i.e., the whole of FR1) that is chicken sequence. The protein sequence was analyzed, not the nucleotide sequence. The number of human sequences analyzed is given at right*.

*ND, not determined. Chicken FR2 differs by only one (SynVH-SD) or two (SynVH-C) amino acids from the human transgene and this it was not possible to unequivocally assess gene conversion*.

*^a^These gene conversion events affected the first 20 bp of exon 2, including three amino acids of the signal peptide and extending to the first three amino acids of FR1*.

*^b^These gene conversion events included the signal peptide changes, but extended further into FR1 beyond the first three amino acids*.

### CDR-H3 Length Diversity and Frequency of Unique CDR-H3 Sequences

To calculate an overall estimate of the diversity in the CDR-H3 repertoires, the number of different CDR-H3 sequences out of the total number of CDR-H3s was determined. For the SynVH-C samples, there were a total of 178,454 different CDR-H3s out of the pool of about 3 × 10^6^ sequences with a recognizable CDR-H3, and for SynVH-SD, there were a total of 60,870 different CDR-H3s out of about 1 × 10^6^ sequences. In both cases, the frequency of unique CDR-H3 sequences represented about 6% of the total.

**Figure 3:**
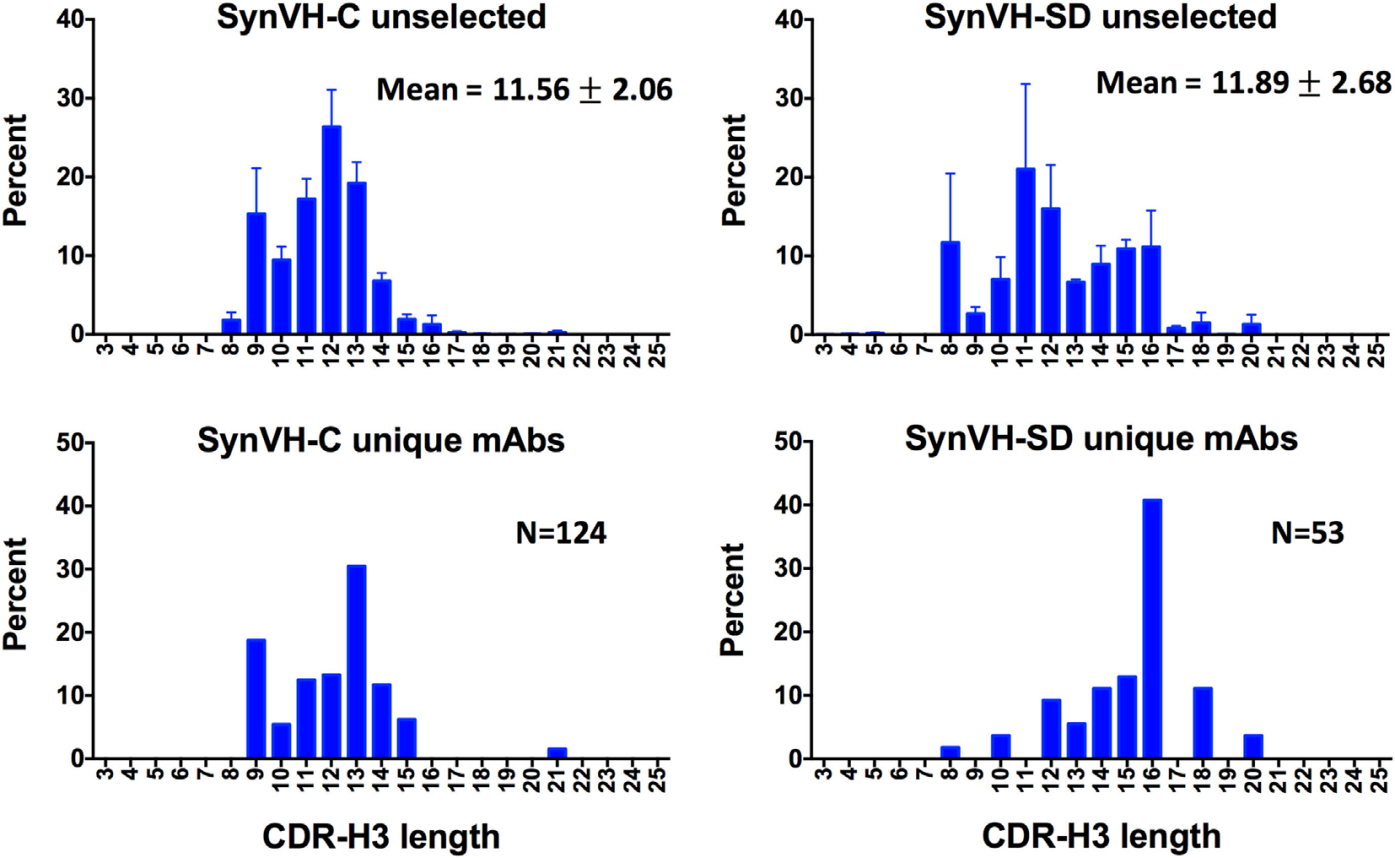
CDR-H3 lengths show a broader distribution in SynVH-SD. CDR-H3 lengths from all of the sequences (“unselected,” top two panels) were calculated based on IMGT positions 105–117 and plotted based on the frequency of each length out of all sequences with a clear CDR-H3 (*N* = 3,099,355 for SynVH-C, *N* = 1,050,398 for SynVH-SD). Error bars indicate bird-to-bird variation; SynVH-C, *N* = 6 birds, SynVH-SD, *N* = 3 birds. Mean and SD are given. Lower two panels show CDR-H3 lengths of the progranulin-specific mAbs.

CDR-H3 length diversity was analyzed in the two genotypes, from IMGT positions 105 to 117. In the SynVH-C sequences, the range was 3–22 amino acids, and the mean was 11.56 ± 2.06 (Figure 3). The CDR-H3 length in the germline of SynVH-C is a fixed length of 11 codons, since it is pre-rearranged, and any variation in length could only be a result of gene conversion or somatic hypermutation that deleted or inserted sequences. These mechanisms were more likely to increase the CDR length, as 55% have lengths longer than 11 residues, yet only 27% of the sequences have a length less than 11. In the rearranging transgene SynVH-SD, the mean CDR-H3 length was slightly longer and the distribution broader (11.89 ± 2.68 amino acids, range 3–21; neither set of length data fits a normal distribution) (Figure 3). Longer CDR-H3s were more frequent in SynVH-SD, with 23% of the CDRs of length 15 residues and above, as compared to 3.6% for SynVH-C. The hypothetical range of lengths in SynVH-SD, with no chewing back of coding sequences, would be 16–23 codons if a single D is used, indicating that most sequences undergo reduction in length either from chewing back during V(D)J recombination or from gene conversion/somatic hypermutation. Although the mAb sequences are limited and only for one antigen, the CDR-H3 lengths were skewed toward the longer lengths for SynVH-SD whereas SynVH-C frequencies seem to match the bulk sequencing (Figure 3, lower panels).

Chicken B cells lack TdT activity (9), so no additional nucleotides can be added during V(D)J rearrangement. In the WT human and chicken repertoires, CDR-H3s tend to be longer than those presented here, with mean CDR-H3 lengths of 15–16 residues, and a normal distribution (17, 29, 30). In WT chickens, the broad range of CDR-H3 lengths is produced by a combination of single D usage (the hypothetical range of lengths with no chewing back would be 20–22 codons if a single D is used), tandem D-D joining, exonucleolytic trimming, and gene conversion (6, 8, 30). In the rearranging SynVH-SD transgene, these mechanisms did not seem to produce the same lengths in the human V regions as in WT chicken antibodies. It is possible that the chicken Ds have been selected in evolution to prefer tandem D-D joins, which are common in chickens and are the mechanism to incorporate paired non-canonical cysteines to form intra-CDR3 disulfide bridges (see below). By contrast, D-D joins are not normally found in the human repertoire (30–32). Although we did not analyze for D usage, the more limited CDR-H3 length found in the human sequences would suggest that D-D joins are not occurring, or if they are, there is selection against longer CDR-H3s produced by D-D joins of human genes in the chicken.

### Amino Acid Content in SynVH-C and SynVH-SD

Amino acid content of the CDRs was analyzed in the SynVH-C and SynVH-SD datasets (Table 4). The chicken V region sequences were removed and only the human sequences were analyzed. These datasets were based on immunized birds so they do not represent the naïve repertoire, but the characteristics of the repertoires produced by the two transgenes may be compared to each other since the immunogen, human PGRN, was the same for all birds. For CDR-H1 and H2 (IMGT definitions were used), all of the sequences were included regardless of length.

**Table 4:** Amino acid distributions in CDRs 1–3.

	CDR1 (IMGT 27–38)		CDR2 (IMGT 56–65)		CDR3 (IMGT 105–117), 12–15AA	
			
	SynVH-C (%)	SynVH-SD (%)	SynVH-C (%)	SynVH-SD (%)	SynVH-C (%)	SynVH-SD (%)
D	5.24	12.16	5.04	4.44	11.50	11.64
E	0.05	0.00	0.59	0.28	2.08	1.02
K	0.12	0.15	0.26	1.16	6.53	5.56
R	1.53	0.57	2.43	1.54	4.51	3.80
H	2.68	0.71	0.60	0.32	0.60	3.46
T	12.11	11.98	12.80	14.07	5.94	4.01
S	17.70	14.85	23.92	23.46	8.05	3.15
N	3.80	3.10	5.02	9.72	6.89	4.84
Q	0.00	0.04	0.06	0.05	0.17	2.29
G	14.74	13.67	23.94	19.35	6.56	4.25*
A	6.39	2.10	6.06	1.56	8.84	9.64
C	0.00	0.03	0.04	0.00	0.05	1.28
P	0.43	0.47	0.51	0.15	1.24	1.80
V	0.56	0.65	2.13	1.03	2.14	10.32*
I	1.20	0.32	14.52	14.85	1.74	1.73
L	0.78	0.37	0.69	0.28	1.26	1.96
M	0.04	0.00	0.06	0.08	0.32	8.25*
F	24.26	24.11	0.17	0.09	14.65*	1.03
Y	8.35	11.43	1.14	3.39	10.90	18.59
W	0	3.27	0.04	4.17	6.05	1.38

IMGT CDR designations were used. For CDR-H3, amino acid frequencies in CDR lengths of 12–15 residues were calculated and averaged. Asterisk (*) indicates those CDR3 residues that are specific to either JH4 (SynVH-C) or JH6 (SynVH-SD). Only the human sequences were analyzed from the six SynVH-C birds (*n* = 5,652) and three SynVH-SD birds (*n* = 3,000).

In CDR-H1, most of the amino acids were represented at similar frequencies in sequences from the two transgenes. One exception was Trp, which was not present in SynVH-C but found at 3.3% in SynVH-SD, all at IMGT position 38. The likely explanation is that Trp is found in three of the SynVH-SD pseudogenes, at position 38, but not in any of the SynVH-C pseudogenes (Figure S1 in Supplementary Material). Similarly, Ala was more prevalent in SynVH-C sequences and reflects the presence of Ala in 15 of the 20 SynVH-C pseudogenes at IMGT position 38. Asp was more frequent in SynVH-SD, again most likely a result of gene conversion from pseudogenes containing Asp (7 out of 16 have at least one Asp in CDR1). Although clonal selection could alter frequencies of relevant amino acids, by increasing the frequency of residues involved in antigen binding to PGRN or having an effect on attributes such as expression level, at least some of the differences in frequencies should be attributable to differences in the rate of production of the changes in the first place.

In CDR-H2, amino acid distribution was also quite similar in the two genotypes, again with the biases usually traceable to residues found in the pseudogene pool. Trp was more frequent in SynVH-SD, and two of the SynVH-SD pseudogenes contain Trp, at IMGT position 58, whereas none of the SynVH-C pseudogenes contains Trp (Figure S1 in Supplementary Material). For Trp to be present in SynVH-C sequences, it could only be caused by somatic hypermutation.

**Figure 4:**
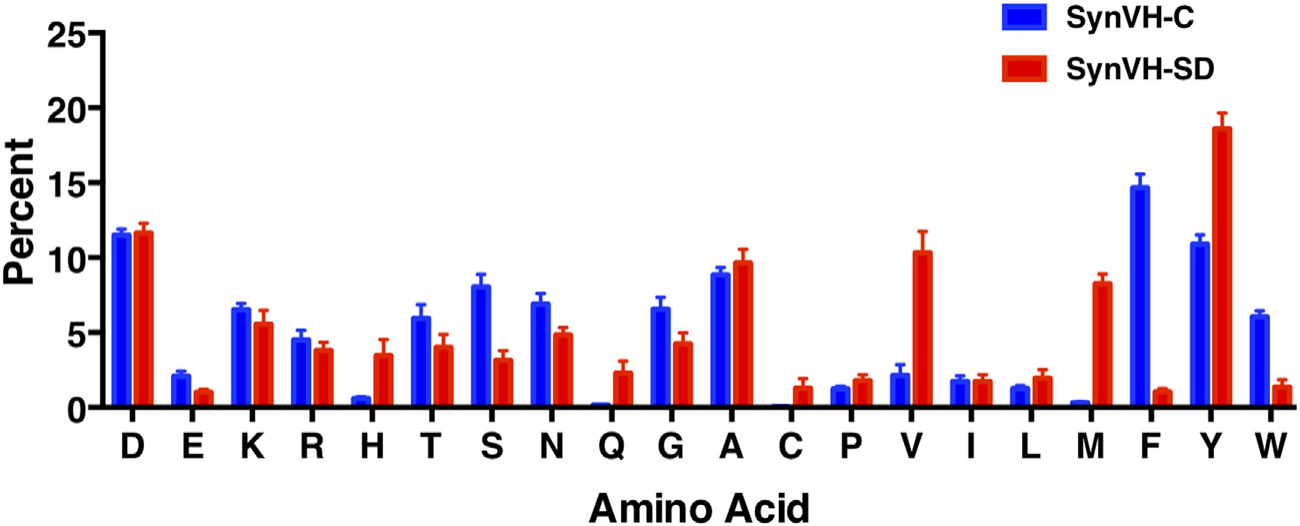
SynVH-C and SynVH-SD amino acid frequencies in CDR-H3 are similar for most amino acids. The frequency of each amino acid out of the total amino acid content in CDR-H3, IMGT positions 105–117. Specific CDR-H3 lengths of 12, 13, 14, and 15 residues from the top 1,000 unique sequences from each bird were used to calculate the frequencies, which were then averaged. Error bars indicate bird-to-bird variation; SynVH-C, *N* = 6 birds, SynVH-SD, *N* = 3 birds.

**Figure 5:**
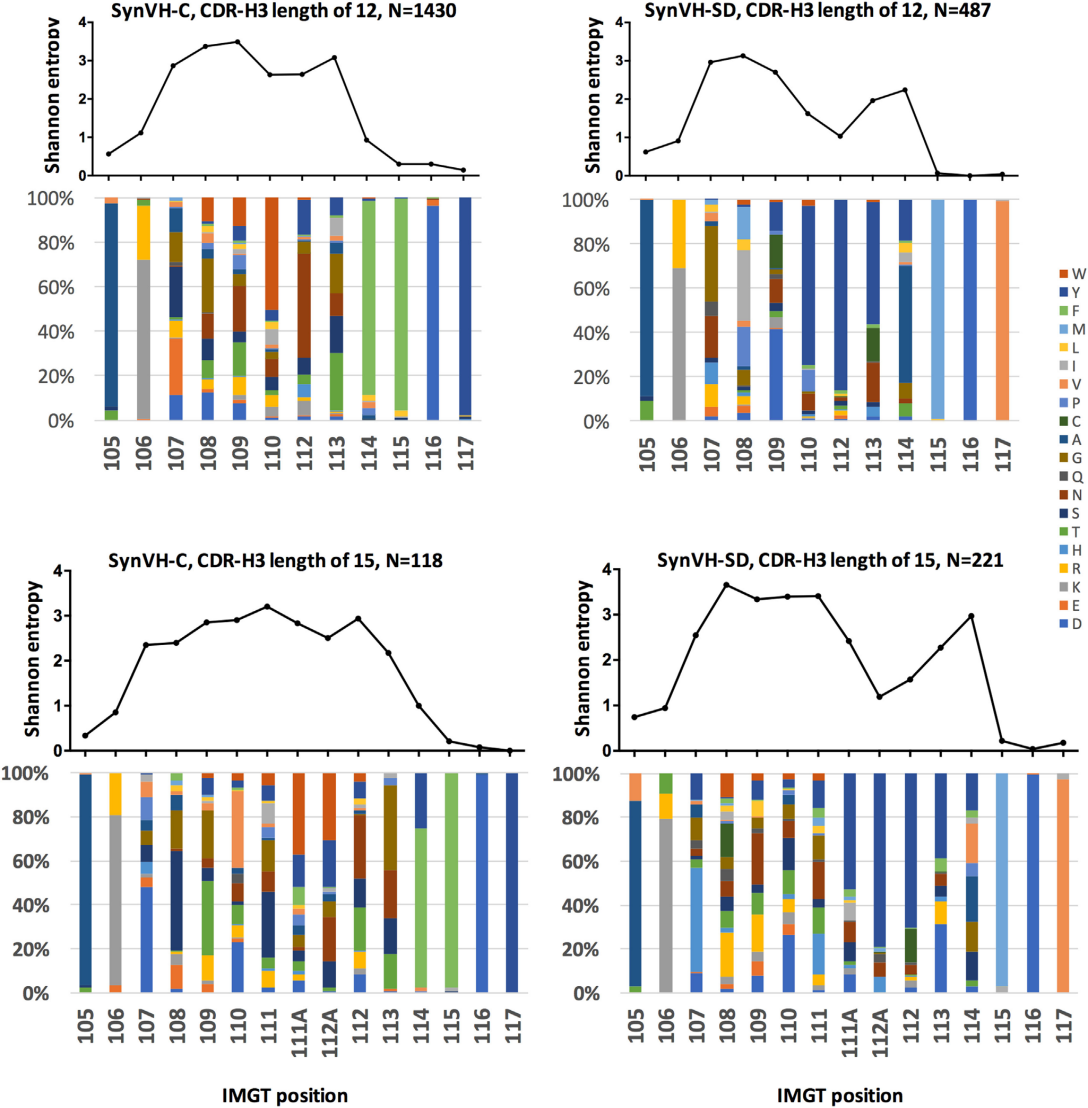
Sequence diversity in CDR-H3 is similar in SynVH-C and SynVH-SD. Shannon entropy and amino acid distribution for each position of CDR-H3 IMGT positions 105–117 are shown for the specific lengths of 12 residues (top two panels) and 15 residues (bottom two panels) for SynVH-C and SynVH-SD. The sequences were drawn from the top 1,000 sequences from each bird; SynVH-C, six birds, SynVH-SD, three birds.

To compare CDR-H3 from SynVH-C and SynVH-SD transgenes, we calculated the average frequencies of each amino acid in CDR-H3s of lengths 12–15 residues (IMGT positions 105–117; Figure 4; Table 4). Although many residues were found at similar frequencies in the two transgenes, there were a few notable exceptions, in particular the amino acids encoded in the germline JH regions. The two transgenes contained different JH regions with quite different sequences. The germline JH region in SynVH-C contained the sequence FDY (JH4), whereas SynVH-SD contained the sequence YYYYYGMDV (JH6). Perhaps not surprisingly, the frequencies of Met and Val were higher in the CDR-H3 sequences from SynVH-SD, and Phe was higher in SynVH-C, correlating with the germline JH sequences in each case. These JH-derived CDR-H3 positions were found to be rarely mutated (Figure 5; see below) so the original germline residues would be expected to influence the overall frequencies in CDR-H3. The frequency of Tyr was high in SynVH-SD, as expected given the string of five Tyr residues in the JH6 region of SynVH-SD, but was also fairly high in SynVH-C, indicating positive selection and the importance of Tyr in CDR-H3 (33, 34). As for the amino acids not found in the relatively unchanging JH regions, only a few differences were observed between the two transgenes. Trp, also a common residue in human CDR-H3, was higher in SynVH-C (6.05%) than SynVH-SD (1.38%). His frequency was much higher in SynVH-SD (3.46%) compared to SynVH-C (0.6%), although the overall frequencies of positively charged amino acids (Lys, Arg, and His) were similar (SynVH-SD 12.8%, SynVH-C 11.6%). Gln was higher in SynVH-SD (2.29%) than in SynVH-C (0.17%). Cys content was also higher in SynVH-SD (see below for details). In CDR-H3, it was not possible to trace any of these differences in amino acid frequency to differences in the residues available in the pseudogene pool (Figure S1 in Supplementary Material). Trp, Gln, and His residues can be found in the CDR-H3 regions of both pseudogene arrays.

To measure the amino acid variability in CDR-H3 of the two transgenes, Shannon entropy was calculated for each position in the subset of CDR3s of length 12–15 codons. Lengths of 12 and 15 residues are shown in Figure 5; 13 and 14 residues gave similar results. In the portion of the CDR-H3 loop mainly contributed by the D segment (positions 107–109 for CDRs of 12 residues and 107–111 for CDRs of 15 residues), diversity was similar for the two transgenes. Thus gene rearrangement and gene conversion alone can lead to similar levels of diversity in the regions not encoded by JH. In the regions that are encoded by JH, there was less diversity in SynVH-SD CDR-H3s, particularly the positions that are 5–7 residues from Trp-118 (such as positions 110, 112, and 113 in CDR-H3s of length 12 in Figure 5). These positions are within the tandem stretch of tyrosines encoded by JH6, and tyrosine content was high at these positions in the sequencing data. However, other positions in the JH6 gene that encode tyrosine were found to be somatically mutated in SynVH-SD birds and could be highly diverse, such as positions 113 and 114. The lack of diversity at positions 112 and 112A may reflect a reduced involvement of these positions in antigen binding, resulting in less selection pressure for somatic mutation. The lack of specific CDR3 sequences in the SynVH-SD pseudogenes may also have been a contributing factor, although the variability in positions 108–111 and 113–114 was not reduced compared to SynVH-C despite those positions also lacking pseudogene donors.

To determine levels of diversity in the FR regions, Shannon entropy was calculated across the entire length of the V region (Figure S2 in Supplementary Material). FRs contained little diversity, in particular FR2. FR variability was somewhat lower in SynVH-SD than in SynVH-C, which may be expected since the pseudogenes in SynVH-C contain some FR changes whereas those in SynVH-SD do not. Any changes in the FRs in SynVH-SD must be from non-templated hypermutation, or potentially from chicken pseudogenes. The FR1 variability observed in SynVH-SD was partly from chicken pseudogenes (as discussed above), but the overall level of variability was still lower than that of SynVH-C in FR1. FR2 had essentially no variability.

The SynVH-C functional V region contains the motif RLF in FR3 (IMGT positions 90–92) that represents a somatic mutation compared to the germline residues QMN found in the VH3-23 gene. Several of the SynVH-C pseudogenes contain the QMN residues in those positions, enabling a reversion to the germline FR3 sequence by gene conversion. This reversion was observed in 94% of the sequences, strongly suggesting that the RLF motif was structurally disfavored and that the QMN residues were selected in the repertoire.

### Hydrophobicity of CDR3

**Figure 6:**
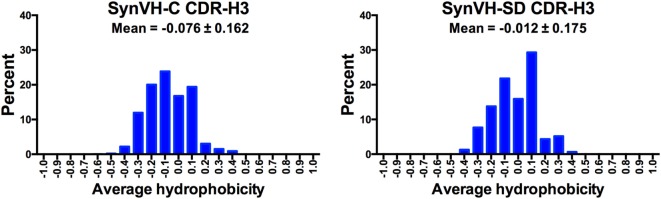
Hydrophobicity of CDR-H3 is similar in SynVH-C and SynVH-SD. Average hydrophobicity based on the normalized Kyte–Doolittle scale (35, 36) of CDR-H3s of lengths 12–15 residues (IMGT positions 105–117) were calculated for the top 1,000 sequences from each bird. The frequencies of each hydrophobicity value (grouped into increments of 0.1) are shown in the graphs.

Average hydrophobicity for each CDR-H3 (IMGT positions 105–117) from SynVH-C and SynVH-SD was calculated, based on the normalized Kyte–Doolittle scale of amino acid hydrophobicities (35, 36). Data from CDR-H3 lengths of 12–15 residues were combined (Figure 6). The mean of these values falls on the hydrophilic side of the scale for both SynVH-C and SynVH-SD, and the range appears similar to that previously reported for human and mouse repertoires (37–39). The mean value for SynVH-SD CDR-H3s is shifted slightly toward the hydrophobic compared to SynVH-C [the difference in the mean of the hydrophobicity values was significant for the two groups (unpaired Kolmogorov–Smirnov test, *p* < 0.0001)].

### Cysteine Content in FRs and CDRs

Low frequencies of non-canonical cysteine residues were found scattered throughout the V regions of SynVH-C and SynVH-SD sequences, either as single unpaired cysteines or pairs of cysteines that could potentially form disulfide bridges (Table 5). A total of 123 unpaired, individual cysteines were found in the 8,652 VH sequences from the two transgenes (Table 5). These residues were found in all FRs and CDRs of both SynVH-C and SynVH-SD, including CDR-H3, except for FR1 of SynVH-C. The frequency of unpaired cysteines was lower than that observed in WT chickens [0.08, 0.13, and 0.17% vs. 2.1, 0.8, and 2.4% in CDR1, FR2, and CDR2 from SynVH vs. WT (17)].

**Table 5:** Non-canonical cysteine content is low in SynVH-C and SynVH-SD birds.

		FR1	CDR1	FR2	CDR2	FR3	CDR3	FR4
SynVH-C	Single Cys	0	2	8	13	5	43	9
	Paired Cys	0	0	0	4 (CDSC)	0	3 (CWNFLC)	0
					2 (CC)			
SynVH-SD	Single Cys	3	5	3	2	2	22	6
	Paired Cys	0	0	0	0	0	34 (CNDYYC)	0
							76 (CYYC)	

*Numbers refer to the number of unique sequences containing a single, unpaired Cys residue or two potentially paired Cys residues in the top 1,000 unique sequences from each bird (only human V regions were analyzed; total, *n* = 5,652 for SynVH-C and 3,000 for SynVH-SD). In parentheses are shown the sequences of the potential loops. The only other potential disulfide loop was a single SynVH-C sequence with Cys residues in FR2 and FR3. In FR2 and FR4, all instances of non-canonical Cys were cases of Trp → Cys changes, which can occur by a single nucleotide substitution. No instances of more than two non-canonical cysteines were observed*.

In CDR-H3, the total cysteine content in SynVH-C sequences was 0.05%, and in SynVH-SD, 1.28% (Table 4), compared to the 1.21% reported in humans (29). The occurrence of cysteine in CDR-H3 is thus similar between human sequences derived from chickens or humans. In addition to the unpaired cysteines, a small number of paired cysteines that could form disulfide bridges was also observed within CDR-H3 of both transgenes (3 sequences in SynVH-C and 110 sequences in SynVH-SD) (Tables 4 and 5). SynVH-C also had six instances of paired cysteines in CDR-H2, but SynVH-SD had none. These paired cysteines form potential disulfide-stabilized loops, but there is very little diversity in the sequences of the loops themselves. Only two unique sequences each from SynVH-C and SynVH-SD were found, indicating that these paired cysteines only occurred rarely and then spread by clonal expansion within their family trees. It was striking that SynVH-SD contained more instances of potential disulfide loops in CDR-H3 than SynVH-C (3.7% of the SynVH-SD sequences contained a potential intra-CDR3 disulfide loop compared to 0.05% of SynVH-C sequences). Although the SynVH-SD transgene had the D cluster which could in principle provide higher cysteine content, the transgene was designed such that the Cys codons normally found in the germline human D2 family members were all mutated to encode Tyr or Trp. In the sequences of the SynVH-SD pseudogenes, the region downstream of the FR3 does contain some Cys codons, so gene conversion (as well as somatic hypermutation) could have added cysteines. The SynVH-SD pseudogene design was based on the germline VH3 family CDRs, and since germline V genes do not contain CDR3, the region downstream of FR3 in the pseudogenes was simply a spacer sequence between pseudogenes. These spacers could provide a potential source of diversity if they are used in gene conversion.

The cysteine content of the human sequences from chickens is low, as in human-derived sequences, which is in sharp contrast to the cysteine content of normal chicken antibodies (17). In WT chickens, 53% of non-selected VH clones contained two non-canonical cysteines in CDR-H3, compared to 1.3% of the human sequences from chickens. These paired cysteines may form small loops that stabilize the antigen-binding structure in chicken CDR-H3s. The cysteines are separated by 2–4 amino acids in the human sequences presented here, whereas in some cases the loops in chicken antibodies can be longer (17). Chicken VH sequences often contain a single cysteine in CDR3 and a second cysteine elsewhere in the VH region, forming a potential disulfide bridge from CDR3 to another part of the V region (Types 3–6 of Wu et al.). No such paired cysteines were observed in the human sequences from the chicken. Only one sequence out of 8,652 from both transgenes had two non-canonical cysteines in different parts of the V region, a single SynVH-C sequence with cysteines in FR2 and FR3. This particular pattern was not seen in chickens (17).

### Comparison of mAbs to NGS Data

The spleen lymphocytes that were the source of the NGS data came from birds that had been immunized and used to produce antigen-specific antibodies. The six SynVH-C and three SynVH-SD birds were immunized with a test immunogen, human PGRN. Panels of antigen-specific mAbs were identified from each bird, by screening single spleen cells in GEM assays (18, 23). GEMs containing B cells secreting anti-PGRN antibodies were picked under the microscope, and variable regions were amplified by single cell RT-PCR and cloned into a single chain Fv-Fc format. The mAbs were confirmed as binding to PGRN by ELISA, and kinetic analysis showed that binding affinities ranged from 0.11 to over 200 nM, including some in the sub-nanomolar category (18). The light chain in all of these birds was provided by a human V-kappa transgene (18). The birds were heterozygous for both heavy and light chain transgenes, with light and heavy chain knockouts on the other alleles. Therefore, only human V region antibodies are produced in the birds.

**Figure 7:**
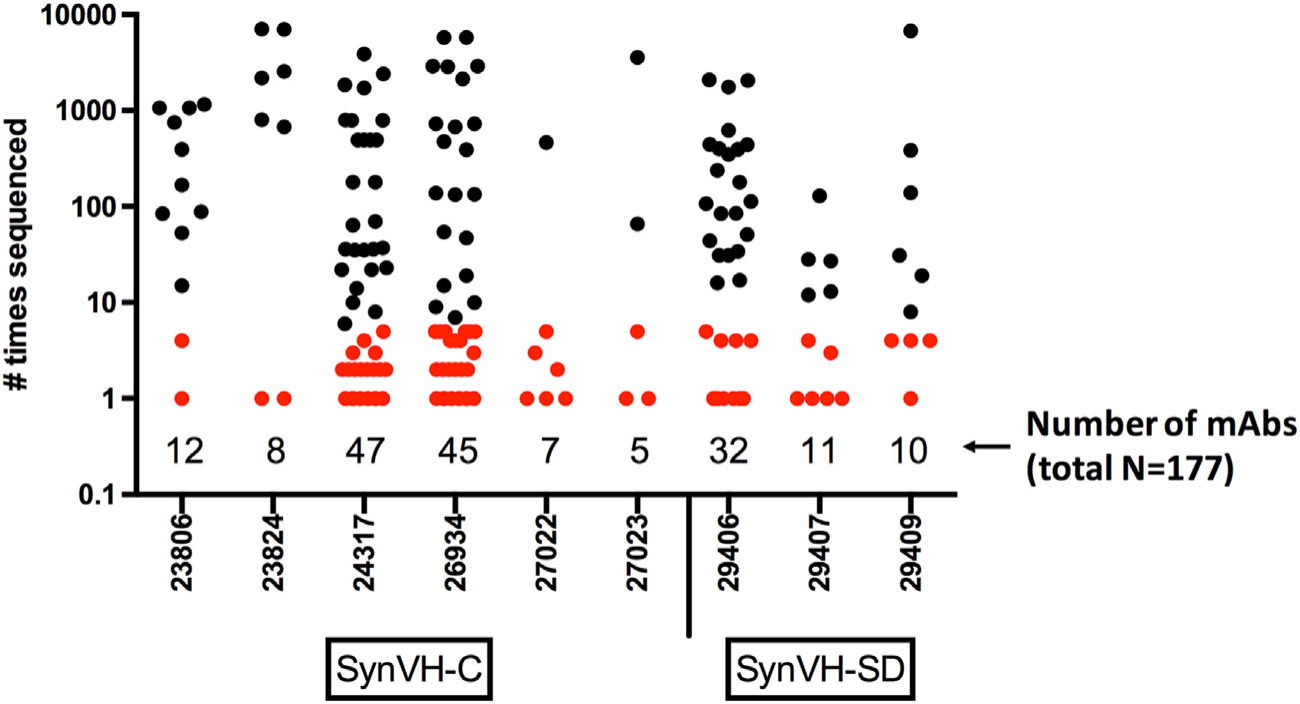
Frequency of mAb sequences in NGS data show that many mAbs identified by gel-encapsulated microenvironment (GEM) screening are rare in the spleen population. Number of times each antigen-specific mAb sequence identified in GEM screens was found in all of the sequencing data from each bird. The mAbs are grouped by bird and transgene, either SynVH-C or SynVH-SD, as indicated. The number of mAbs from each bird is indicated below the plots. mAbs that were sequenced five or fewer times in the whole dataset are colored red, showing that rare mAbs are found in the GEM screens.

The nucleotide sequences of the mAbs were determined, and the unique VH sequences of the antigen-specific mAbs from each individual bird were compared to the NGS data from the same bird (total, 177 mAbs). The number of times each mAb was sequenced in the NGS data is shown in Figure 7. All 177 mAb sequences were found in the NGS data, with varying degrees of matching. 79 of the mAbs (45%) were sequenced five times or fewer in the NGS data, and 37 were only sequenced once, indicating that GEM screens can readily identify rare mAbs. If one were to select mAbs based on sequence data alone, one would likely miss these rare mAbs. Others were found multiple times, up to about 7,000 times, for a total of about 84,000 sequences that could be matched to the set of 177 mAb sequences. Out of the entire NGS dataset (5.3 million unique sequences), the mAb sequences represented about 1.6% of the sequences. About one-third (63/177) of the mAb sequences matched perfectly to a unique peptide sequence in the NGS data, whereas the rest had some changes relative to the closest match. These changes probably reflect the fact that the NGS data represent a snapshot of the spleen, at most about 10^6^ B cells (on average, the total number of sequences from each bird that were obtained), whereas the mAbs were derived from a GEM screen of a larger sample size, about 7 × 10^6^ B cells. The sequence from a single cell picked in a GEM screen might be related to sequences in a family tree in the NGS data although the exact sequence was not found in the NGS data. Sequence errors introduced by reverse transcription or library preparation could also be a factor.

## Discussion

The chicken gene conversion/somatic hypermutation system has evolved to produce a diverse repertoire that is capable of conferring protective immunity on its host. The lack of diversity from combinatorial mechanisms does not seem to be a limitation for the CDR-H3 repertoire. When we introduced two transgenes encoding human variable regions, one which cannot undergo rearrangement but is forced to generate its sequence diversity purely through gene conversion and/or somatic hypermutation, and the other which undergoes rearrangement and has the choice of all human Ds to produce combinatorial diversity, the levels of amino acid diversity were similar. Because our dataset came from immunized birds, there could be some bias toward specific residues from the immunization, but the two transgenes behaved similarly despite having different modes of diversity generation. In CDRs 1 and 2, and the FRs, it was possible to correlate amino acid frequencies to residues found in the human pseudogenes in both transgenes, but for CDR3, it was not. It would be interesting to compare the pre-rearranged, JH4-containing transgene with a rearranging JH4 transgene, which would allow a more direct comparison of the amino acid diversity across the entire CDR-H3 and would remove the potential bias of the string of tyrosines in the JH6 used in the rearranging transgene here.

The range and mean of CDR-H3 lengths were also quite similar in both the pre-rearranged and rearranging transgenes. It is striking that the mean CDR-H3 lengths were so similar in the two transgenes given that the rearranging transgene in principle could produce a much wider range from variation in D usage. The lack of TdT in chickens limits the lengths to what is encoded by the D genes, or what could be inserted by gene conversion, which could explain the shorter lengths seen here compared to the normal human repertoire. Gene conversion alone may not be able to produce as wide a range of CDR-H3 lengths as rearrangement with TdT activity (as in humans), or rearrangement with D-D joins (as in WT chickens). It is curious that SynVH-SD did not seem to produce the range of lengths that were theoretically possible by D-D joins or even single D rearrangements. It would be interesting to determine CDR-H3 lengths produced in early B cell development in SynVH-SD, before further diversification and selection, to know if even longer CDR-H3s are initially produced by D-D joins, and then removed from the repertoire by negative cellular selection. Constraints on CDR-H3 length could come from pairing with the particular V-kappa light chain in the OmniChickens, potentially unfavorable structural attributes of such human D-D sequences, or unknown *cis*-acting sequences that promote D-D joins in chicken that were not included in the human SynVH-SD construct. Even though the range and mean CDR-H3 lengths were similar, SynVH-SD did contain a significant proportion of longer CDR-H3s (15 residues and above) that could represent an advantage in an antibody discovery program, as there may be more opportunity for added functionality, such as broader epitope coverage, agonistic or antagonistic functions, and kinetics. It has recently been shown that cow antibodies with ultra-long (~60 amino acid) H3 domains can be potent neutralizers across many clades of HIV, possibly due to their ability to bind conserved, but occluded, epitopes on the virus (40).

Structures that are commonly found in the chicken, such as non-canonical disulfide bridges, were extremely rare in the human sequences. The human germline sequences and pseudogenes that are available during repertoire development do not encode such structures, so there is no obvious mechanism to produce them. To obtain high-affinity human antibodies in the chicken, these structures are not necessary since the mAbs compared in this study had a range of affinities down to 0.11 nM, and epitope coverage similar to that in WT chickens (18). These data are supported by the observation that mAbs from WT chickens recognizing novel epitopes do not require a disulfide bridge although they are frequently present (41).

Selection for functional sequences was clearly in effect for the germline VH3-23 signal peptide in SynVH-SD and for the QMN motif in FR3 of SynVH-C. The germline VH3-23 gene contains a lysine residue in the signal peptide, which was mutated (to Ile) in the human somatically derived SynVH-C signal peptide. Multiple mechanisms came into play to mutate the SynVH-SD signal peptide sequence in chickens, including gene conversion at the 5′ end of the V gene. This result shows that gene conversion of chicken pseudogenes could act on the human functional V, when it could provide a useful purpose. Despite the ability for gene conversion from chicken pseudogenes to act on the human V region, very little gene conversion of the human FRs was observed in the bulk sequencing, and in the mAb sequences, no chicken gene conversion was found at all. From the standpoint of the gene conversion mechanism, the chicken pseudogenes would be at a disadvantage because of their physical distance from the human functional V and their reduced level of homology (5, 11, 13), which could explain the lack of gene conversion by chicken pseudogenes. From a functional standpoint, the diversity that is achievable by the human pseudogenes and somatic hypermutation is extensive enough to enable antibodies capable of binding antigen, so in general there is no selective pressure driving further mutation by the chicken pseudogenes.

Our data were derived from immunized birds, not from the pre-immune repertoire, which could have influenced such attributes as amino acid frequencies and CDR-H3 lengths. However, we expect that the majority of the sequences were not from antigen-specific B cells responding to the immunogen. We can estimate the frequency of antigen-specific cells in the population from single B cell screening in the GEM assay (23). From a starting population of about 6 × 10^4^ spleen lymphocytes that are secreting sufficient antibody to be detected in GEMs, we typically identify 10^2^–10^3^ cells secreting antigen-specific antibody, a frequency of about 1%. In the NGS dataset, the frequency of the antigen-specific mAb sequences was 1.6% of the total, which correlates with the frequency in GEMs and further suggests that most of the sequences were from cells not producing PGRN-specific antibodies. Despite the fact that the majority of the sequences may not be demonstrably PGRN-specific, the immunization process and cellular selection could still influence the outcome, and it would be worthwhile to compare to the IgM repertoire in non-immunized birds.

Almost half of the mAb sequences that had been identified in GEM screens were relatively rare, found five times or fewer in the NGS data. The mAb sequences presented here had affinities ranging down to sub-nanomolar (18). Antibody discovery on the basis of frequencies in sequence data may miss some of these rare sequences, since there would be no way to know the significance of the rare sequence changes without the functional connection provided by screening directly for binding mAbs, followed by sequencing.

The different strategies employed by chickens and humans to produce their CDR-H3 repertoires are reflected in the results from our human transgenes. V(D)J recombination is dispensable for the generation of a diverse sequence repertoire in chickens, as the gene conversion/somatic hypermutation mechanisms seem to be fully capable of generating a highly diverse repertoire of human sequences when the V genes are placed into the chicken heavy chain locus. However, it remains to be seen whether the mechanisms for producing length diversity in chickens can be applied to the human genes.

## Ethics Statement

This study was carried out in accordance with the recommendations of the Animal Care and Use Protocol for the use of OmniChickens of the Institutional Animal Care and Use Committee (IACUC) of Ligand Pharmaceuticals (formerly Crystal Bioscience). The protocol was approved by the Ligand Pharmaceuticals IACUC.

## Author Contributions

PL prepared amplicons for sequencing, analyzed the data, and wrote the paper. JM and KC performed immunizations, screened for mAbs, and characterized identified mAbs. WH planned experiments and edited the paper.

## Conflict of Interest Statement

The authors are employees of Ligand Pharmaceuticals, which has issued patents and patents pending regarding the OmniChickens described in this work.
