# Supplementary material for: V(D)J Rearrangement Is Dispensable for Producing CDR-H3 Sequence Diversity in a Gene Converting Species
Source: Front Immunol. 2018 Jun 11;9:1317. doi: 10.3389/fimmu.2018.01317 (PMC6008532; doi:10.3389/fimmu.2018.01317)
Supplement: Supplementary file 1 [file image_1.PDF]

SynVH-C

```
HuVH     DVQLVESGGGVVRPGESLRLSCAASGFTFTNYDMSWVRQAPGEGLEWVSAISGSGDTTYYADSVKGRFTISRDNSKNTLYLRLFSLRAEDTAIYYCAKGTWNTFFDYWGLGTLVTVSS
SynVH48  E.........L.Q..G.......V...P.SS.A.................S....AI.........................................DMVPYREGGNSGYYYGMDVD
SynVH49  .............................SSSAR........K............GS.F.......................................AWELLHIQPDAAPGFSGAH.
SynVH38  E.........L.Q..G...........S.SS.A................I..V..TS........................................RDRGGIAAQDTHGVHVSRAAL
SynVH39  .............................S..G.T....P..K......G.R...GGA......R....V...........QMN........V.....DSTVSWYRALFQH.PGFME.
SynVH40  E.........L.Q..G..............S.A................S...N.GS.........................................DLGYYYDSSGDY.GSRGMDV
SynVH41  .............................SS.G................G..A..NSA.......................................EDSY..GWTEYFQHIDGKGMR
SynVH42  .............................SS..................G...R.GS.........................................PEGGRGAAAGPFDAFDISPG
SynVH43  .............................DI.A.........K...........AYS................S.......QMN........V.....DGIAAGHTRARGSAYPGEMD
SynVH44  .............................S.FA.T.......K.P.......H..GS..S......................................RDRRSSIAARL..YYYYYGM
SynVH45  .............................SSHA....................N.VSA........................................DPFGYSNYGFDYFAS.SPGF
SynVH46  .............................ST.A................R.....GS.D..E...................................RSKEDYDILTGYNLQYYYYGM
SynVH47  .............................SS.AV........K...........A.S...................S....QMN........V.....SGRFGDLWEVYYYG.DVAYR
SynVH50  ...........................S.SS.A.N..............G...R.GS........................................RDFRYYDAINNYGILVTWTAR
SynVH51  .............................RS.A.N..............L.....ESI.......................QMN........V....NREQLW.SSGETYYYYYGMDV
SynVH52  .............................S..A.T..............V..S.............................................DR.WGTDSDA.V.QHSGLIV
SynVH53  E...LD....L.Q..G..T..........S..A.G..............G..A..GS.........................................ENNRHGGVSTFG.V.TSPHG
SynVH54  .............................SS.V................D.R....S...S.....................................AAAHYYDILT.YFPSYYYYG
SynVH55  .............................SI.A................V.I....RIH..............S.N.....QMN........V.....ISGDIVVVPAAFHDVAPGLN
SynVH56  E.........L.Q..G......T......R..A.P..............TL.DN.GS...........................................ASRNSLLPYNP.FGGVIH.
SynVH57  .............................SSDA................S.....LN.D.......................................VG.ELPYGVSPGFGGLAHGP
```

SynVH-SD

```
HuVDJ    EVQLLESGGGLVQPGGSLRLSCAASGFTFSSYAMSWVRQAPGKGLEWVSAISG--SGGSTYYADSVKGRFTISRDNSKNTLYLQMNSLRAEDTAVYYCAKEYNWNDYYYYYGMDVWGQGTTVTVSS
SynVH58  ................................W..................KQ--D.SEK........................................NPGFGG--VIHSPHGEIRA
SynVH59  .............................DD.....................W--NS..I........................................DVAPGL--NGGLR.PIPGE
SynVH60  ..............................D.Y...................S--..STI........................................ARTGLT--GTRVHLHL.RL
SynVH61  ................................D..................GT--A.DTY........................................GGSHRH--HRVASSSASGK
SynVH62  ..............................NAW..................KSKTD..T.........................................GMEREV--MTIKPEC.PTP
SynVH63  .............................DD.G..................NW--N............................................PPTPTC--P-TPRPPLTEPM
SynVH64  ................................S...................S--.SSYI........................................ILVTWT---AR.H.FICIWE
SynVH65  ....................................................Y--D.SNK........................................RAPIS---PRGLWAPLNP.A
SynVH70  ............................V..NY..................-Y--.............................................RALISQ--PGWSVTPLSAE
SynVH71  ....................................................S--N............................................RCT.----TRVPLPSISP..A
SynVH72  ..............................DHY.................TRNKANSYT.........................................SPGLGS--LSLPRNPGPTL
SynVH73  ..............................GS...................RSKANSYA.........................................QGGPSK--GR.TRIPLPSM
SynVH74  ................................W..................NS--D.S..........................................GSMTE.--CPPMPRWTE.R
```

Alignments of designed human pseudogenes compared to the functional V sequences in each construct are shown. For SynVH-C, diverse CDR-H1, 2 and 3 sequences were obtained from the NIH EST database queried with the human VH3-23 gene. The germline VH3-23 framework sequence was included in some of the pseudogenes, whereas others match the functional VH sequence in the SynVH-C construct (HuVH, top line) which is a somatically-derived sequence with 9 changes relative to the germline VH3-23 gene.

For SynVH-SD, CDR-H1 and 2 were derived from the human VH3 family members, and placed on the germline VH3-23 framework scaffold. No specific CDR-H3 sequences were included; the sequences shown are the spacers placed between pseudogenes. The V region shown on the top line is a theoretical sequence derived from recombining germline VH3-23, D1 and JH6 genes.
