# Supplementary material for: V(D)J Rearrangement Is Dispensable for Producing CDR-H3 Sequence Diversity in a Gene Converting Species
Source: Front Immunol. 2018 Jun 11;9:1317. doi: 10.3389/fimmu.2018.01317 (PMC6008532; doi:10.3389/fimmu.2018.01317)

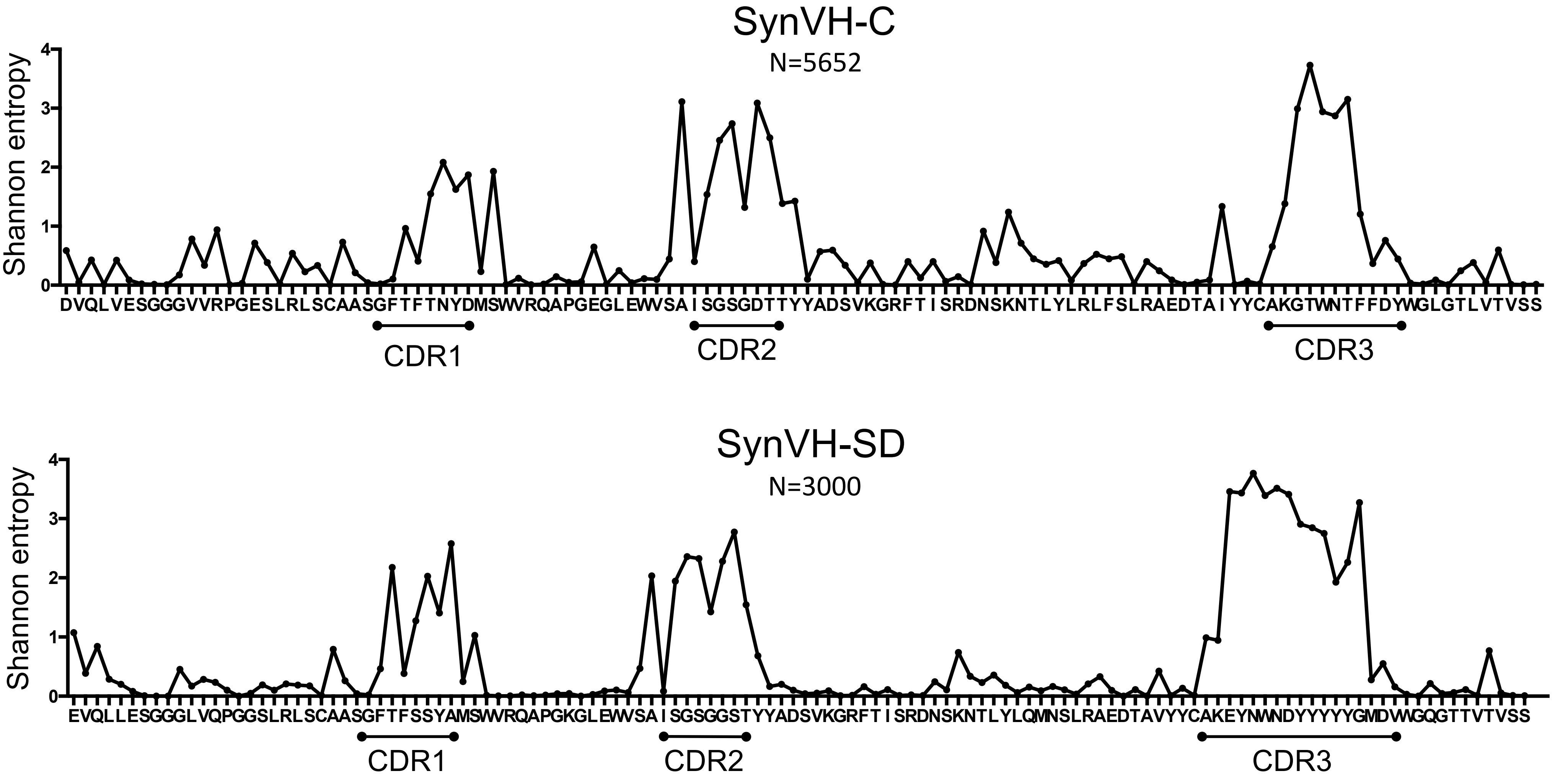

Shannon entropy for all of the aligned V region sequences from SynVH-C and SynVH-SD. The top 1000 sequences from each bird were included. CDR designations (IMGT) are indicated.

Wu-Kabat variability

SynVH-C
N=5652
(huV only)

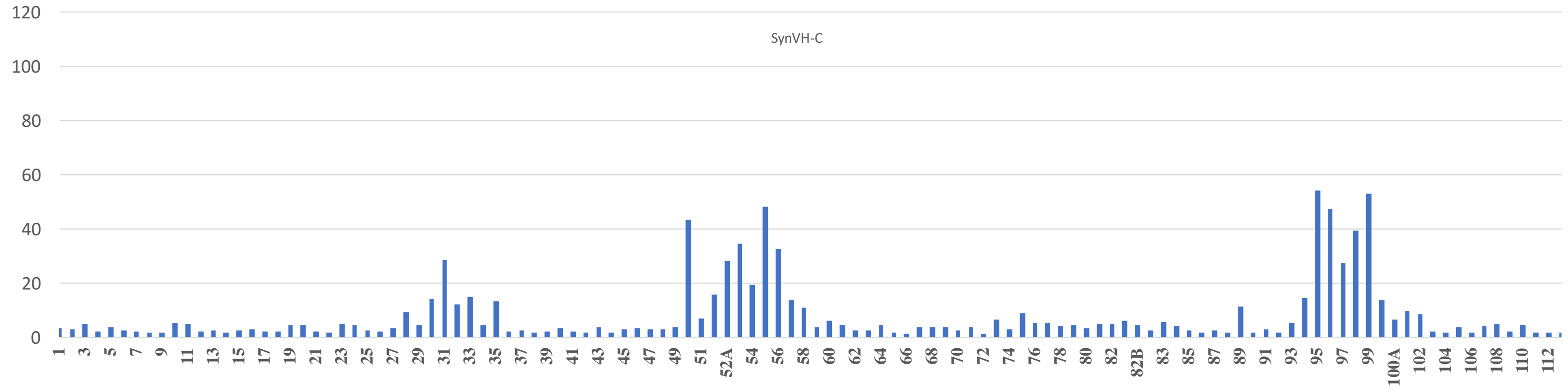

SynVH-SD
N=3000
(huV only)

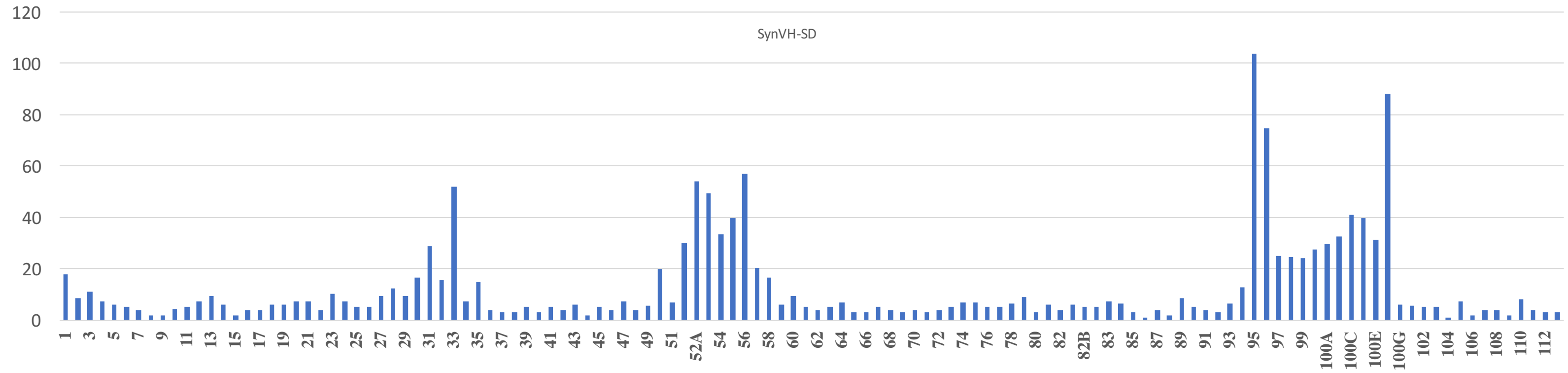

Supplement: Supplementary file 2 [file image_2.PDF]
